# Impact of Vitamin E supplementation on vascular function in haptoglobin genotype stratified diabetes patients (EVAS Trial): a randomised controlled trial

**DOI:** 10.1038/s41387-020-0116-7

**Published:** 2020-04-27

**Authors:** Rinkoo Dalan, Liuh Ling Goh, Chien Joo Lim, Aruni Seneviratna, Huiling Liew, Cherng Jye Seow, Lian Xia, Daniel E. K. Chew, Melvin K. S. Leow, Bernhard O. Boehm

**Affiliations:** 1 Tan Tock Seng Hospital, Singapore, Singapore; 2 Lee Kong Chian School of Medicine, Nanyang Technological University, Singapore, Singapore; 3 Yong Loo Lin School of Medicine, National University Singapore, Singapore, Singapore; 4 Duke-NUS Medical School, Singapore, Singapore

**Keywords:** Diabetes complications, Nutrition

## Abstract

**Aims:**

Vitamin E (Vit-E) may preferentially improve cardiovascular risk in haptoglobin 2-2 (Hp2-2) genotype diabetes individuals. We studied the impact of Vit-E supplementation on vascular function in diabetes individuals stratified by haptoglobin genotype in Singapore.

**Methods:**

In this 24-week, double blind, placebo-controlled RCT, we recruited 187 subjects (101 Hp2-2, 86 non-Hp2-2). Intervention: alpha-tocopherol-400 IU. Primary Outcome: Change in EndoPAT-derived reactive-hyperaemia index (RHI) and augmentation index (AIx); Secondary Outcomes: Pulse-Wave velocity (Sphygmocor-PWV), carotid intima media thickness (CIMT), inflammation (hsCRP), derivatives of reactive-oxygen metabolites (dROMs), biological antioxidant-potential (BAPs), HbA1c, LDL-C, HDL-C and oxidised LDL-C (ox-LDL).

**Results:**

Overall, with Vit-E supplementation no significant change in RHI, PWV, CIMT, hsCRP, dROMS, BAPs, HDL-C and HbA1c was observed (*p* > 0.05); an increase in LDL-C with concomitant decrease in ox-LDL, and incidentally increase in eGFR was observed (*p* < 0.05). No interaction effect with haptoglobin genotype was seen for all outcomes (*p* > 0.05). Subgroup analysis: In the non-Hp-2-2 group, Vit-E supplementation led to a higher EndoPAT-derived AIx, accompanied by higher LDL and ox-LDL concentrations (*p* < 0.05); Hp2-2 group: Vit-E supplementation led to higher eGFR when compared to the non-Hp2-2 group (exploratory) (*p* < 0.05). We observed an interaction effect for baseline haptoglobin concentration (threshold > 119 mg/dl) with intervention in terms of increased EndoPAT-derived AIx in the Hp > 119 mg/dl group whereas no change in the group with Hp ≤ 119 mg/dl.

**Conclusion:**

Vit-E supplementation did not show any preferential benefit or deleterious effect on vascular function in Hp2-2 diabetes subjects in Singapore. A possible deleterious effect of an increase in arterial stiffness in individuals with Hp > 119 mg/dl was observed. Future studies should consider personalisation based on baseline Hp concentrations in patients with T2DM rather than just Hp2-2 genotype to evaluate impact on the detailed lipid pathways, cardiac and renal physiology. The impact of ethnic differences needs to be explored in greater details.

## Introduction

Type 2 Diabetes Mellitus (T2DM) leads to a high disease burden due to vascular complications^1^. The dysmetabolic state in T2DM fuels low-grade inflammation and oxidative stress, thereby compromising vascular reactivity and function^2^.

Haptoglobin (Hp), an α2-glycoprotein with strong free haemoglobin (Hb) affinity, serves as an acute phase reactant to inflammation and functions as an antioxidant in chronic metabolic disorders. Hp is polymorphic with three common isoforms: Hp1-1, Hp2-2 and Hp2-1. Individuals with haptoglobin 2-2 (Hp2-2) genotype have lower Hp concentrations and reduced Hb binding capacity^3^. As well, Hb-Hp2-2 complexes have lower binding affinity for the CD163 scavenger receptor expressed by M2 macrophages which results in a lower haem iron clearance rate and reduced anti-inflammatory cytokine response by macrophages compared with Hb-Hp1-1 and Hb-Hp1-2 complexes^3–5^.

Among individuals with Hp2-2 genotype and diabetes, there is accentuated demand for a high capacity Hp system because of higher erythrocyte lysis coupled with an inefficient macrophage scavenging system^3,4^. Natural history analysis from prospective cohorts has shown that Hp2-2 T2DM individuals have a higher risk of cardiovascular complications^4,5^. Vitamin E (Vit-E) has been classified as a potent antioxidant due to its ability to scavenge lipid radicals and terminate oxidative stress reactions. Animal and post-hoc interventional analysis from Vit-E trials have shown that it has preferential benefits in the Hp2-2 genotype individuals in terms of cardiovascular complications in diabetes^6,7^.

In one double-blind crossover study done in 20 Hp2-2 subjects (10 Vit-E/10 placebo) with T2DM, 8 weeks of daily Vit-E 400 IU supplementation showed significant improvement in peripheral vascular function measured as forearm blood flow and forearm vascular resistance^8^. However, since no comparison with non-Hp2-2 genotype was made, it is not conclusive in terms of whether the positive response is confined to Hp2-2 individuals. The small sample size of this study also beckons for confirmatory studies before any clinical recommendations.

The distribution of the *Hp* gene frequencies follows Hardy–Weinberg equilibrium in the multi-ethnic population of Singapore with the expected prevalence of Hp2-2 to be around 30–40%^9^. We conducted a randomised controlled trial (RCT) to study the impact of Vit-E supplementation on inflammation, oxidative stress, vascular function in diabetes individuals stratified by haptoglobin genotype. To the best of our knowledge, this is the first real world RCT to monitor the entire process of inflammation, oxidative stress, vascular reactivity and stiffness under longer-term, optimum dose Vit-E supplementation in individuals stratified by haptoglobin genotype.

## Methods

### Study population and setting

Consecutive T2DM patients were recruited from a tertiary diabetes centre. The inclusion criteria for randomisation was clinical diagnosis of T2DM, age 21–80 years, stable diabetes, blood pressure (BP) and hyperlipidaemia medications (a 25% dose adjustment was allowed) in the last 3 months, glycated haemoglobin (HbA1c) 6.4 to 10%, BP < 180/120 mm Hg and current non-smokers. The exclusion criteria was inability to give informed consent, pregnancy, hospitalisation for any condition or recent infections (last 2 weeks), eGFR < 20 mL/min/1.73 m^2^, concomitant warfarin, immunosuppressive agents, corticosteroids, orlistat, cholestyramine or Vit-E supplementation, Vit-E allergy, current smoking, malignancies or rheumatological conditions.

The study was conducted according to the principles of the Declaration of Helsinki. Written informed consent was obtained from all participants. Ethics approval was obtained from the local Institutional Review Board [Domain Specific Review Board (DSRB Ref: 2014/00236)]. Clinical trials authorisation was obtained from the national regulator (CTC1500174). The trial was registered at clinicaltrials.gov NCT02776397.

### Study design and randomisation

We conducted a 24-week randomised, double blind, placebo-controlled, parallel group study stratified by Hp2-2 genotype status. The randomisation schedule was created by independent statisticians. A blocked randomisation schedule was employed, in blocks of 10, based on a 1:1 allocation ratio.

Patients were randomly allocated to either Vit-E or placebo group using a centralised interactive password-protected, web-based service which allocated a unique patient trial number corresponding to the medication label numbers. After randomisation, the study drug was dispensed according to the serial numbers generated and allocation was blinded to both the patients and the study personnel.

The intervention medication used was Vit-E 200 IU (International units) in powder form (50%) incorporated into hard gelatin capsules and placebo pills which consisted of magnesium stearate (white colour granules) only. The Vit-E formulation was the natural alpha-tocopherol which occurs in the RRR-configuration. The medications were manufactured by Beacon’s pharmaceuticals, Singapore. They were labelled as consecutive serial numbers and blinded by two independent study personnel at the site. Each subject was prescribed to take two tablets every day (total 400 IU of Vit-E daily in the intervention arm) for a period of 24 weeks. The study nurses, investigators and patients were hence completely blinded of the assignment.

Compliance was assessed by the percentage of prescribed pills ingested. A compliance rate of more than 70% was considered satisfactory. The last patient was recruited on 1st January 2018 and completed follow-up on 1st June 2018.

### Measurements

All outcome measurements were performed by the study team masked with respect to Vit-E intake and biochemical outcome data at both baseline and follow-up visits.

The physical measurements included height, weight and BP.

Plasma haptoglobin concentrations were measured by turbidimetry. Serum creatinine concentrations were measured by Jaffe Reaction. Estimated GFR (eGFR) was calculated using the Chronic Kidney Disease Epidemiology Collaboration (CKD-EPI) equation^10^.

Hp genotyping was performed using TaqMan-based real-time polymerase chain reaction^11^. Alpha-tocopherol concentrations were measured in plasma samples using the alpha-tocopherol (TCPa) BioAssay^TM^ ELISA Kit (USBiological, 028903).

The primary outcome measurement was the reactive-hyperaemia index (RHI-EndoPAT)^12^ and augmentation index (AI@75 beats/min)^13^ measured using the EndoPAT 2000 (Itamar Medical Ltd, Israel).

The secondary outcome measurements included: (1) Oxidative stress markers measured as derivatives of reactive-oxygen metabolites (dROMS) and the biological antioxidant (BAPs) (FREE Carrio Duo; Diacron International) using measurement kits (Wismerll Co Ltd, Tokyo)^14^; Oxidised LDL(ox-LDL) were measured in plasma samples using the Mercodia Oxidised-LDL ELISA kit (Mercodia AB, 10-1143-01)^15^. (2) Inflammation measured as high-sensitivity C-reactive protein (hsCRP) by turbidimetry. (3) HbA1c was measured by immunoturbidimetric assay (Beckman Coulter Synchron LX^®^20, Brea, CA, USA). (4) Fasting lipids were measured using standard coupled enzymatic methods and LDL cholesterol was calculated by the Friedewald equation. (5) We used the SphygmoCor Xcel device to estimate the aortic artery stiffness using the carotid to femoral pulse-wave velocity (PWV)^16^. (6) Carotid ultrasonography was performed using a 5.0- to 13.0-MHz multi-frequency high-resolution linear transducer probe (GE Logiq P5) by two trained operators. The carotid artery intima media thickness (CIMT) measurement procedure followed the Mannheim CIMT consensus recommendations^17^. Pilot examination on 23 volunteers showed acceptable limits of inter- and intra-user agreement with a coefficient of variance of ±0.1 by Bland–Altman analysis for all of above measurements.

### Statistical analysis

We calculated a minimal sample size of 80 in each *Hp* genotype stratum based on: 5% type I error; 80% power; the assumption that Vit-E have a standardised effect size (mean difference/pooled-standard deviation) of 0.5, on each risk marker; two sample *t* test with equal variance; and a 15% drop out rate. If we assume a mean difference on RHI of 0.25 units with corresponding standard deviation (SD) of 0.3 would yield a standardised effect size of 0.25/0.3 = 0.83 as seen in another study^18^. A per-protocol analysis for all patients who came back for final completion visit was planned.

Data on baseline demographic and clinical variables have been summarised by Hp genotype and treatment allocation group. Descriptive statistics are summarised as count (n) and percentage of non-missing by category for categorical data and mean, standard deviation (SD) or median, interquartile range (IQR) for continuous data.

An analysis of the effect of Vit-E supplementation versus placebo was initially performed to assess the effect of intervention in the overall population and then an interaction test was performed to see the effect of haptoglobin genotype (Hp2-2 vs non-Hp2-2) on each outcome. Subsequent comparisons by Hp genotype and Vit-E use were made using independent sample *t* test (normally distributed) or Mann–Whitney U test (skewed) for continuous data, and Chi-square or Fisher’s exact test for categorical data. We analysed the final measurements in the Hp2-2 and non-Hp2-2 groups adjusted for baseline alpha-tocopherol concentrations using ANCOVA.

Correlation between absolute Hp concentrations with vascular measurements and blood markers were initially assessed using Spearman rank-order method. Subsequent multivariable models were built to adjust for confounding variables to study independent associations.

Statistical analysis was performed using IBM SPSS statistics version 19.0 and Stata (version 13.1, College Station, TX: StataCorp LP), significance tests were 2-sided at 5% significance level.

## Results

### Participants

**Fig. 1:**
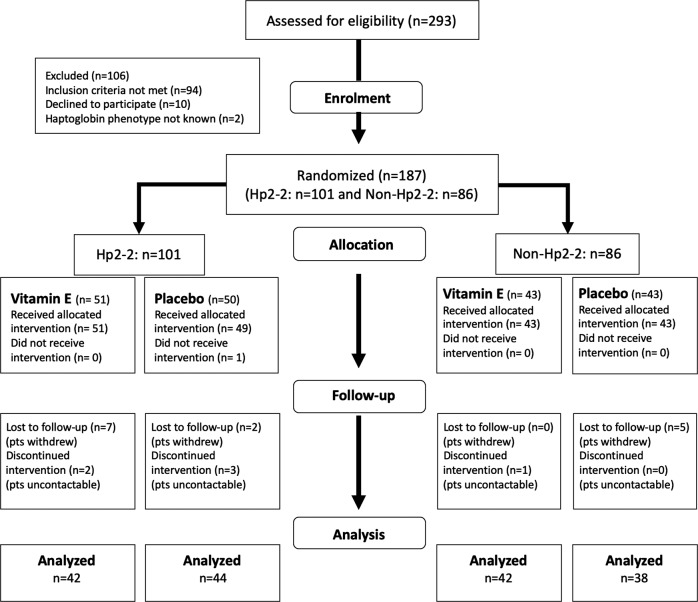
Consort diagram. Flow of subjects in the study is shown using the CONSORT flow diagram.

Between 17 June 2016 and 31 December 2017, we screened 293 consecutive patients with T2DM visiting the Diabetes Centre. Out of 293 participants screened, we randomised 187 participants (Hp 2-2: 101 participants, non-Hp 2-2: 86 participants) to Vit-E supplementation or placebo stratified by Hp genotype. As per protocol analysis, 86 subjects in the Hp2-2 group (42: Vit- E group, 44: placebo group) and 80 subjects in the non-Hp2-2 group (42: Vit-E group, 38-placebo group) were analysed. (see CONSORT diagram, Fig. 1).

The baseline characteristics in all four groups are summarised in Table 1a, b. Demographic and baseline biochemical variables were well balanced between the four groups. The Hp2-2 group consisted of a greater percentage of Indians when compared to the non-Hp2-2 group, Hp concentrations were lower and LDL-C concentrations were higher in the Hp2-2 group when compared to the non-Hp2-2 group (*p* < 0.05; Table 1b). There were no significant differences in terms of inflammation, oxidative stress, alpha-tocopherol concentrations, RHI-EndoPAT, AIx, PWV and CIMT at baseline. Approximately 13 (7%) had baseline stable ischaemic heart disease (IHD). There was no significant difference in baseline IHD in the four study groups. None of the patients had a history of peripheral vascular disease or stroke. The distribution of the haptoglobin genotypes in the non-Hp2-2 group was Hp2-1: 63/86 (33.87%), Hp1-116/86 (8.60%) and Hp1-del: 7/86 (3.76%).

**Table 1:** (a) Baseline variables in the different randomisation arms and by the Haptoglobin genotypes. (b) Baseline variables by Hp genotype.

	Total	Overall		Hp2-2 group		Non-Hp2-2 group	
		Vitamin E	Placebo	Vitamin E	Placebo	Vitamin E	Placebo
(a) *Baseline variables in the different randomisation arms and by the Haptoglobin genotypes*							
Number (%)	166	84 (50.6)	82 (49.4)	42 (25.30)	44 (26.51)	42 (25.30)	38 (22.89)
Age years, mean (SD)	56 (10)	55 (10)	57 (10)	54 (9)	56 (11)	57 (11)	59 (9)
*Gender*							
Female, n (%)	83 (50.00)	41 (49.40)	42 (50.60)	23 (54.76)	24 (54.55)	18 (42.86)	18 (47.37)
Male, n (%)	83 (50.00)	43 (51.81)	40 (48.19)	19 (45.24)	20 (45.45)	24 (57.14)	20 (52.63)
*Ethnicity*							
Chinese, n (%)	103 (62.05)	56 (54.37)	47 (45.63)	21 (50.00)	22 (50.00)	35 (83.33)	25 (65.79)
Malay, n (%)	24 (14.46)	11 (45.83)	13 (54.17)	5 (11.90)	7 (15.91)	6 (14.29)	6 (15.79)
Indian, n (%)	39 (23.49)	17 (43.59)	22 (56.41)	16 (38.10)	15 (34.09)	1 (2.38)	7 (18.42)
Duration of DM years, Median (IQR)	10 (13)	10 (13)	10.5 (12.3)	9.5 (14)	10 (10.5)	10 (11)	11 (14)
Duration of Hypertension years, Median (IQR)	6.5 (13)	3 (12)	8.5 (15.3)	2 (10)	8 (12)	9 (13)	10.5 (17)
Duration of Hyperlipidaemia years, Median (IQR)	7 (11)	6 (12)	8.5 (10.5)	3 (9)	8 (9)	8 (14)	9 (16)
Number of patients with known stable IHD, N(%)	13 (7)	6 (7)	7 (8.5)	4 (9.5)	3 (6.8)	2 (4.8)	4 (10)
*Physical measurements*							
BMI Kg/m^2^, mean (SD)	27.37 (4.94)	27.36 (4.92)	27.39 (5.01)	27.44 (5.38)	28.08 (5.24)	27.26 (4.47)	26.58 (4.65)
Waist circumference cm, mean (SD)	93.81 (10.55)	93.51 (10.04)	94.13 (11.12)	92.48 (9.31)	95.32 (11.14)	94.52 (10.73)	92.73 (11.06)
Systolic BP (Peripheral) mmHg, mean (SD)	134 (15)	135.64 (14.86)	132.32 (14.93)	134 (12)	133 (16)	137 (17)	131 (14)
Diastolic BP (Peripheral) mmHg, mean (SD)	76 (8)	76.64 (8.52)	74.93 (8.12)	77 (9)	76 (8)	76 (9)	74 (8)
HbA1c %, mean (SD)	7.92 (1.17)	7.83 (1.13)	8.02 (1.21)	7.81 (1.26)	8.13 (1.42)	7.84 (1.00)	7.90 (0.91)
*Lipid parameters*							
Total cholesterol mmol/L, Median (IQR)	4.1 (1.2)	4.2 (1.2)	3.9 (1.0)	4.25 (1.3)	4.05 (0.95)	4.2 (0.89)	3.75 (0.89)
LDL cholesterol mmol/L, Median (IQR)	2.3 (0.9)	2.4 (1.0)	2.1 (0.8)	2.5 (1.2)	2.3 (1.0)	2.3 (0.6)	2.0 (0.6)
HDL cholesterol mmol/L, Median (IQR)	1.1 (0.2)	1.1 (0.3)	1.1 (0.2)	1.1 (0.3)	1.1 (0.2)	1.0 (0.3)	1.1 (0.2)
Triglycerides mmol/L, Median (IQR)	1.3 (1.0)	1.3 (1.1)	1.3 (0.8)	1.3 (1.2)	1.3 (0.9)	1.35 (1.2)	1.2 (0.8)
Creatinine μmol/L, mean (SD)	75.02 (21.08)	74.29 (21.56)	75.75 (20.72)	73.91 (21.14)	74.56 (20.58)	74.66 (22.27)	76.97 (21.07)
eGFR ml/min/1.73 m^2^, mean (SD)	89.57 (21.50)	91.71 (23.92)	87.43 (18.70)	92.91 (20.30)	88.47 (19.63)	90.48 (27.39)	86.37 (17.90)
Urine ACR mg/mmol, Median (IQR)	1.55 (3.4)	1.30 (3.10)	1.90 (3.55)	1.3 (3)	1.9 (5.55)	1.2 (3)	2.4 (3.5)
*Haematological parameters*							
Haptoglobin mg/dL, mean (SD)	116.12 (50.68)	116.55 (54.55)	115.68 (46.74)	107.61 (57.21)	103.45 (42.81)	125.47 (50.85)	129.84 (47.62)
Ferritin μg/L, Median (IQR)	72 (112)	77 (100)	59 (106)	66 (76)	63.5 (119)	101 (133)	54.5 (100)
Transferrin g/L, mean (SD)	2.48 (0.36)	2.47 (0.38)	2.50 (0.34)	2.53 (0.42)	2.48 (0.34)	2.40 (0.33)	2.51 (0.33)
Fe Concentration μmol/L, mean (SD)	13.67 (4.65)	14.17 (4.96)	13.10 (4.23)	14.02 (5.76)	12.68 (4.76)	14.31 (4.04)	13.55 (3.57)
Haemoglobin g/dL, mean (SD)	13.17 (1.42)	13.20 (1.54)	13.16 (1.32)	13.16 (1.49)	13.00 (1.42)	13.25 (1.62)	13.42 (1.11)
Alpha-tocopherol concentration μg/ml, Median (IQR)	38.67 (32.80)	39.92 (37.58)	38.23 (31.12)	31.55 (27.63)	32.97 (34.05)	42.12 (37.67)	39.78 (22.98)
*Vascular markers*							
hsCRP mg/L, Median (IQR)	1.45 (3.1)	1.2 (3.4)	1.7 (2.7)	2.1 (3.8)	1.9 (2.4)	1 (2.2)	1.3 (3.2)
Oxidative Stress index: dROMS, Median (IQR)	271 (73)	271 (85)	274.5 (63)	287 (109)	277 (62)	263 (59)	267.5 (59)
Oxidative Stress index: BAPs uM, Median (IQR)	2221 (327)	2220 (341)	2223 (315)	2225 (362)	2253 (283)	2206 (254)	2189.5 (384)
CIMT: Average of left and right mm, mean (SD)	0.65 (0.128)	0.65 (0.14)	0.65 (0.12)	0.627 (0.099)	0.659 (0.129)	0.674 (0.162)	0.645 (0.110)
CIMT: Maximum of left and right mm, mean (SD)	0.793 (0.152)	0.79 (0.16)	0.79 (0.14)	0.764 (0.112)	0.798 (0.154)	0.823 (0.195)	0.787 (0.132)
EndoPAT: Endothelial function - LnRHI, mean (SD)	0.670 (0.250)	0.68 (0.24)	0.66 (0.26)	0.689 (0.246)	0.655 (0.280)	0.677 (0.236)	0.658 (0.244)
EndoPAT: Augmentation index at 75bpm %, mean (SD)	17.7 (15.0)	18.1 (15.5)	17.4 (14.6)	18 (16.5)	17.8 (15.1)	18.1 (14.6)	17.0 (14.0)
Sphygmocor Systolic BP (Central) mmHg, mean (SD)	121 (14)	122 (13)	120 (14)	122 (12)	122 (14)	123 (14)	118 (14)
Sphygmocor Diastolic BP (Central) mmHg, mean (SD)	79 (9)	80 (10)	78 (9)	80 (10)	79 (8)	79 (10)	77 (9)
Sphygmocor Pulse Wave Velocity m/s, mean (SD)	8.34 (1.74)	8.39 (1.66)	8.30 (1.84)	8.20 (1.47)	8.38 (1.88)	8.57 (1.81)	8.21 (1.80)
SphygmoCor: Augmentation index %, mean (SD)	29.7 (10.1)	29.1 (9.7)	30.3 (10.6)	29.4 (10.9)	30.4 (11.2)	28.7 (8.4)	30.2 (9.7)
Oxidised LDL IU/L, Median (IQR)	53.44 (19.49)	55.8 (21.5)	51.7 (20.5)	56.30 (21.18)	51.71 (17.37)	55.17 (21.78)	51.63 (23.41)
*Medications: Diabetes*							
Sulfonylurea, n (%)	79 (47.59)	44 (55.70)	35 (44.30)	25 (59.52)	18 (40.91)	19 (45.24)	17 (44.74)
Metformin, n (%)	153 (92.17)	77 (50.33)	76 (49.67)	38 (90.48)	40 (90.91)	39 (92.86)	36 (94.74)
DPP-IV, n (%)	18 (10.84)	9 (50.00)	9 (50.00)	2 (4.76)	7 (15.91)	7 (16.67)	2 (5.26)
Long acting Insulin, n (%)	62 (37.35)	32 (51.61)	30 (48.39)	16 (38.10)	12 (27.27)	16 (38.10)	18 (47.37)
Short acting Insulin, n (%)	40 (24.10)	16 (40.00)	24 (60.00)	6 (14.29)	11 (25.00)	10 (23.81)	13 (34.21)
Mixtard, n (%)	8 (4.82)	5 (50.00)	4 (50.00)	2 (4.76)	3 (6.82)	2 (4.76)	1 (2.63)
Pioglitazone, n (%)	2 (1.20)	1 (50.00)	1 (50.00)	1 (2.38)	1 (2.27)	0	0
SGLT-inhibitors, n (%)	13 (7.83)	6 (46.15)	7 (53.85)	4 (9.52)	3 (6.82)	2 (4.76)	4 (10.53)
*Medications: Hyperlipidaemia*							
Statin, n (%)	129 (77.71)	62 (48.06)	67 (51.94)	32 (76.19)	35 (79.55)	30 (71.43)	32 (84.21)
Fibrate, n (%)	17 (10.24)	11 (64.71)	6 (35.29)	5 (11.90)	2 (4.55)	6 (14.29)	4 (10.53)
Ezetimibe, n (%)	2 (1.20)	1 (50.00)	1 (50.00)	0	1 (2.27)	1 (2.38)	0
*Medications: Hypertension*							
ACE-Inhibitor, n (%)	51 (30.72)	21 (41.18)	30 (58.82)	12 (28.57)	15 (34.09)	9 (21.43)	15 (39.47)
Angiotensin Receptor Blocker, n (%)	57 (34.34)	24 (42.11)	33 (57.89)	15 (35.71)	16 (36.36)	9 (21.43)	15 (44.74)
Beta blocker, n (%)	31 (18.67)	16 (51.61)	15 (48.39)	7 (16.67)	8 (18.18)	9 (21.43)	7 (18.42)
Calcium channel blocker, n (%)	36 (21.69)	17 (47.22)	19 (52.78)	8 (19.05)	12 (27.27)	9 (21.43)	7 (18.42)
Diuretics, n (%)	11 (6.63)	5 (45.45)	6 (54.55)	2 (4.76)	2 (4.55)	3 (7.14)	4 (10.53)
Aspirin, n (%)	31 (18.67)	11 (35.48)	20 (64.52)	8 (19.05)	11 (25.00)	3 (7.14)	9 (23.68)
Other antiplatelet, n (%)	6 (3.61)	3 (50.00)	3 (50.00)	3 (7.14)	2 (4.55)	0	1 (2.63)

	Total	Non-Hp2-2	Hp2-2	*p* value
(b) *Baseline variables by Hp genotype*				
Number (%)	166	80 (48.20)	86 (51.80)	
Age years, mean (SD)	56 (10)	55 (10)	58 (10)	0.071
*Gender*				
Female, n (%)	83 (50.00)	36 (43.37)	47 (56.63)	0.214
Male, n (%)	83 (50.00)	44 (53.01)	39 (46.99)	
*Ethnicity*				
Chinese, n (%)	103 (62.05)	60 (58.25)	43 (41.75)	<0.001*
Malay, n (%)	24 (14.46)	12 (50.00)	12 (50.00)	
Indian, n (%)	39 (23.49)	8 (20.51)	31 (79.49)	
Duration of DM years, mean (SD)	12 (8)	12 (9)	11 (7)	0.201
Duration of Hypertension years, mean (SD)	8.5 (9)	7 (8)	10 (10)	0.016*
Duration of Hyperlipidaemia years, mean (SD)	9 (9)	11 (10)	8 (7)	0.033*
Number of patients with known stable IHD, N(%)	13 (7)	6 (7.5)	7 (8.1)	0.878
*Physical measuremen*ts				
BMI Kg/m^2^, mean (SD)	27.37 (4.94)	26.94 (4.54)	27.77 (5.29)	0.282
Waist circumference cm, mean (SD)	93.81 (10.55)	93.67 (10.86)	93.94 (10.33)	0.871
Systolic BP (Peripheral) mmHg, mean (SD)	134 (15)	134 (16)	134 (14)	0.879
Diastolic BP (Peripheral) mmHg, mean (SD)	76 (8)	75 (8)	76 (8)	0.291
HbA1c %, mean (SD)	7.92 (1.17)	7.87 (0.95)	7.98 (1.35)	0.573
*Lipid parameters*				
Total cholesterol mmol/L, mean (SD)	4.20 (0.92)	4.09 (0.94)	4.30 (0.90)	0.142
LDL cholesterol mmol/L, Median (IQR)	2.3 (0.9)	2.2 (0.7)	2.4 (1.1)	0.036*
HDL cholesterol mmol/L, Median (IQR)	1.1 (0.2)	1.1 (0.3)	1.1 (0.2)	0.856
Triglycerides mmol/L, Median (IQR)	1.3 (1.0)	1.3 (1.0)	1.3 (1.0)	0.404
Creatinine μmol/L, mean (SD)	75.02 (21.08)	74.24 (20.73)	75.82 (21.56)	0.653
eGFR ml/min/1.73 m^2^, median (IQR)	89.6 (25.6)	88.7 (22.5)	93.6 (26.5)	0.248
Urine ACR mg/mmol, Median (IQR)	1.6 (3.4)	1.6 (3.70)	1.5 (4.30)	0.646
*Haematological parameters*				
Haptoglobin mg/dL, mean (SD)	116.12 (50.68)	127.55 (49.09)	105.49 (50.11)	0.005*
Ferritin μg/L, Median (IQR)	72 (112)	81 (119)	65 (101)	0.123
Transferrin g/L, mean (SD)	2.48 (0.36)	2.46 (0.34)	2.51 (0.38)	0.347
Fe Concentration μmol/L, mean (SD)	13.67 (4.65)	13.97 (3.83)	13.40 (5.32)	0.478
Haemoglobin g/dL, mean (SD)	13.17 (1.42)	13.33 (1.40)	13.07 (1.44)	0.438
Alpha-tocopherol concentration μg/ml, Median (IQR)	38.67 (32.80)	41.89 (33.07)	31.98 (34.00)	0.062
*Vascular markers*				
hsCRP mg/L, Median (IQR)	1.45 (3.1)	1.05 (2.78)	1.95 (3.45)	0.088
Oxidative Stress index: dROMS, Median (IQR)	271 (73)	266 (62)	278.5 (74)	0.124
Oxidative Stress index: BAPs uM, Mean (SD)	2218.12 (280.74)	2220.47 (289.43)	2234.33 (273.19)	0.441
CIMT: Average of left and right mm, mean (SD)	0.65 (0.13)	0.66 (0.14)	0.64 (0.12)	0.4
CIMT: Maximum of left and right mm, mean (SD)	0.793 (0.152)	0.81 (0.17)	0.78 (0.14)	0.311
EndoPAT: Endothelial function - LnRHI, mean (SD)	0.670 (0.250)	0.67 (0.24)	0.67 (0.26)	0.926
EndoPAT: Augmentation index at 75bpm %, mean (SD)	17.7 (15.0)	17.6 (14.3)	17.9 (15.8)	0.908
Sphygmocor Systolic BP (Central) mmHg, mean (SD)	121 (14)	121 (14)	122 (13)	0.699
Sphygmocor Diastolic BP (Central) mmHg, mean (SD)	79 (9)	78 (10)	80 (9)	0.352
Sphygmocor Pulse Wave Velocity m/s, mean (SD)	8.34 (1.74)	8.40 (1.81)	8.30 (1.69)	0.69
SphygmoCor: Augmentation index %, mean (SD)	29.7 (10.1)	29.5 (9.1)	30.0 (11.0)	0.75
Oxidised LDL IU/L, Median (IQR)	55.92 (21.65)	56.71 (23.33)	55.20 (20.08)	0.655

*DM* Diabetes mellitus, *BMI* body mass index, *BP* blood pressure, *eGFR* estimated glomerular filtration rate by CKD-EPI formula, *ACR* albumin creatinine ratio, *LDL-C* Low density lipoprotein cholesterol, *HDL-C* high density lipoprotein cholesterol, *Fe* iron, *hsCRP* highly sensitive c-reactive protein, *dROMS* derivatives of reactive-oxygen species, *BAPs* Biological antioxidant potential, *CIMT* carotid artery intima media thickness, *LnRHI* log reactive-hyperaemia index. *p < 0.05.

Based on pill counting, approximately 59/84 (70%) patients in the Vit-E group and 62/82 (76%) in the placebo group were compliant to the intervention medications and consumed more than 70% of the pills prescribed. Minor side effects of nausea, abdominal bloating, constipation, headache and lethargy were reported in both placebo and Vit-E groups. Most patients were able to continue the medications without any problems.

### Primary analysis

We first analysed the effect of Vit-E supplementation in the whole group. Vit-E supplementation did not result in an improvement in the primary outcomes of RHI-EndoPAT and RHI-EndoPAT-derived augmentation index (AI@75bpm) (*P* > 0.05; Table 2a). Amongst the secondary outcomes, no significant difference was seen in the physical measurements of BMI, waist circumference, blood pressure; haematological parameters, inflammation, oxidative stress, PWV and CIMT. Interestingly, Vit-E supplementation resulted in higher total cholesterol and LDL cholesterol but lower ox-LDL when compared to placebo group (*P* < 0.05; Table 2a). Incidentally, we found that the eGFR was higher in the intervention group when compared to the placebo group (*P* < 0.05). This outcome would be considered exploratory as this was not a pre-specified outcome. The interaction test did not show any interaction effect by Hp genotypes with Vit-E supplementation on the outcomes.

**Table 2:** (a) Final measurements at 6 months with comparisons between Vitamin E and placebo group and results of interaction test with haptoglobin genotype. (b) Subgroup analysis of final measurements at 6 months with comparisons between haptoglobin genotype and Vitamin E and placebo group.

Variables	Overall				Interaction test				
	Vitamin E	Placebo	Diff (95% CI)	*p* value	Hp 2-2		Non-Hp 2-2		*p* value
					Vitamin E	Placebo	Vitamin E	Placebo	
(a) *Final measurements at 6 months with comparisons between Vitamin E and placebo group and results of interaction test with haptoglobin genotype*									
Number (%)	84 (50.6)	82 (49.4)			42 (25.30)	44 (26.51)	42 (25.30)	38 (22.89)	
Time from baseline to completion visit days, Median (IQR)	176.00 (12.00)	177.00 (14.00)		0.329^b^	176.50 (13.00)	177.00 (14.00)	175.50 (12.00)	177.00 (14.00)	
*Physical measurements*									
BMI Kg/m^2^, mean (SD)	27.55 (4.93)	27.23 (5.03)	−0.32 (−1.84, 1.21)	0.684^a^	27.79 (5.33)	27.96 (5.36)	27.30 (4.54)	26.39 (4.54)	0.482^f^
Waist circumference cm, mean (SD)	92.97 (10.27)	94.10 (11.18)	1.12 (−2.16, 4.41)	0.500^a^	91.78 (9.94)	95.17 (11.11)	94.17 (10.57)	92.85 (11.30)	0.160^f^
Systolic BP (Peripheral) mmHg, mean (SD)	133.27 (15.05)	129.62 (13.51)	−3.66 (−8.04, 0.73)	0.102^a^	133.21 (13.63)	128.92 (14.08)	133.33 (16.51)	130.42 (12.96)	0.758^f^
Diastolic BP (Peripheral) mmHg, mean (SD)	74.18 (8.66)	71.95 (6.90)	−2.23 (−4.63, 0.18)	0.069^a^	74.61 (8.30)	72.78 (7.51)	73.75 (9.09)	70.99 (6.07)	0.701^f^
*Metabolic parameters*									
HbA1c %, mean (SD)	7.75 (1.19)	7.99 (1.37)	0.25 (−0.15, 0.64)	0.219^a^	7.79 (1.26)	8.29 (1.67)	7.70 (1.13)	7.65 (0.80)	0.169^f^
Total cholesterol mmol/L, Median (IQR)	4.10 (1.08)	3.90 (1.30)	−0.20 (−0.52, 0.12)	*0.034^b^	4.30 (1.23)	4.20 (1.10)	4.10 (0.93)	3.60 (1.25)	0.303^f^
LDL cholesterol mmol/L, Median (IQR)	2.40 (0.83)	2.10 (1.00)	−0.30 (−0.52, −0.08)	*0.014^b^	2.40 (1.13)	2.30 (0.80)	2.30 (0.70)	1.90 (0.75)	0.569^f^
HDL cholesterol mmol/L, Median (IQR)	1.10 (0.40)	1.10 (0.30)	0.00 (−0.08, 0.08)	0.628^b^	1.10 (0.23)	1.10 (0.28)	1.10 (0.40)	1.10 (0.30)	0.960^f^
Triglycerides mmol/L, Median (IQR)	1.40 (1.15)	1.20 (1.10)	−0.20 (−0.51, 0.11)	0.348^b^	1.35 (0.75)	1.30 (1.25)	1.40 (1.30)	1.10 (0.90)	0.069^f^
*Renal parameters*									
Creatinine μmol/L, mean (SD)	71.69 (19.97)	78.59 (25.01)	6.91 (−1.13, 14.94)	0.092^a^	66.52 (18.89)	78.27 (24.62)	77.03 (19.95)	78.94 (25.82)	0.225^f^
eGFR ml/min/1.72m^2^, mean (SD)	93.07 (17.33)	84.62 (21.44)	−8.46 (−15.35, −1.57)	*0.017^a^	97.49 (16.75)	85.18 (22.97)	88.51 (16.99)	84.02 (20.06)	0.263^f^
ACR mg/mmol, Median (IQR)	1.50 (3.45)	2.20 (4.30)	0.70 (−0.81, 2.21)	0.147^b^	1.25 (3.18)	1.75 (5.45)	2.15 (4.63)	2.90 (4.25)	0.484^f^
*Haematological parameters*									
Haptoglobin mg/dL, mean (SD)	109.67 (52.03)	115.76 (48.14)	6.09 (−9.28, 21.46)	0.435^a^	98.98 (44.44)	101.50 (39.12)	120.36 (57.20)	132.26 (52.66)	0.536^f^
Ferritin μg/L, Median (IQR)	72.00 (83.00)	58.50 (93.00)	−13.50 (−40.01, 14.01)	0.322^b^	57.50 (70.00)	58.50 (107.00)	79.00 (94.00)	58.00 (90.00)	0.213^f^
Transferrin g/L, mean (SD)	91.42 (77.24)	85.26 (78.16)	−6.16 (−29.98, 17.66)	0.610^a^	2.51 (0.40)	2.46 (0.36)	2.35 (0.41)	2.46 (0.40)	0.210^f^
Fe Concentration μmol/L, mean (SD)	12.89 (5.30)	12.75 (4.48)	−0.15 (−1.86, 1.57)	0.865^a^	12.90 (4.87)	11.72 (4.68)	12.89 (5.73)	13.81 (4.07)	0.226^f^
Alpha-tocopherol, μg/ml,Median (IQR)	44.02 (44.32)	43.20 (36.74)	−1.02 (−13.54, 11.51)	0.682^b^	38.25 (38.04)	39.22 (33.56)	52.04 (46.30)	45.53 (44.10)	0.829^f^
*Vascular markers*									
hsCRP mg/L, Median (IQR)	1.45 (2.95)	1.50 (2.50)	0.05 (−0.76, 0.76)	0.982^b^	1.90 (3.08)	1.4 (2.95)	1.20 (1.93)	1.65 (2.43)	0.356^f^
dROMS, Median (IQR)	275.00 (85.00)	275.50 (115.00)	0.50 (−18.87, 22.87)	0.768^b^	293.50 (87.00)	270.5 (120.00)	266.50 (85.00)	280.50 (115.0)	0.226^f^
BAPs uM, Median (IQR)	2231.00 (311.0)	2191.50 (354.0)	−39.50 (−125.63, 51.63)	0.836^b^	2218.0 (315.00)	2192.5 (393.00)	2252.5 (326.0)	2186.5 (418.0)	0.458^f^
CIMT: Average of left and right mm, mean (SD)	0.67 (0.14)	0.67 (0.12)	−0.004 (−0.04, 0.04)	0.822^a^	0.66 (0.12)	0.67 (0.13)	0.69 (0.15)	0.67 (0.12)	0.531^f^
CIMT: Maximum of left and right mm, mean (SD)	0.82 (0.16)	0.81 (0.15)	−0.01 (−0.06, 0.04)	0.737^a^	0.80 (0.14)	0.81 (0.15)	0.84 (0.18)	0.82 (0.15)	0.558^f^
EndoPAT: LnRHI, mean (SD)	0.66 (0.27)	0.65 (0.25)	−0.02 (−0.10, 0.06)	0.690^a^	0.64 (0.26)	0.68 (0.23)	0.69 (0.28)	0.61 (0.26)	0.128^f^
EndoPAT: Augmentation index 75bpm%,mean (SD)	20.23 (17.52)	15.89 (12.03)	−4.34 (−8.94, 0.26)	0.064^a^	19.90 (16.66)	18.48 (13.23)	20.55 (18.53)	12.89 (9.80)	0.184^f^
Pulse Wave Analysis: Systolic BP mmHg, mean (SD)	118.98 (14.42)	115.32 (12.18)	−3.66 (−7.75, 0.44)	0.079^a^	119.62 (12.64)	115.95 (12.32)	118.33 (16.13)	114.58 (12.13)	0.983^f^
Pulse Wave Analysis: Diastolic BP mmHg,mean (SD)	77.81 (9.76)	75.70 (8.38)	−2.11 (−4.91, 0.68)	0.137^a^	77.83 (8.68)	76.59 (8.51)	77.79 (10.84)	74.66 (8.22)	0.508^f^
Pulse Wave Velocity m/s, mean (SD)	8.48 (1.58)	8.41 (1.64)	−0.07 (−0.56, 0.42)	0.776^a^	8.34 (1.56)	8.51 (1.72)	8.62 (1.61)	8.29 (1.55)	0.330^f^
SphygmoCor: Augmentation index %, mean (SD)	29.71 (13.77)	29.26 (9.96)	−0.46 (−4.15, 3.24)	0.808^a^	30.85 (9.07)	29.48 (9.77)	28.60 (17.21)	29.00 (10.31)	0.637^f^
Oxidised LDL IU/L, Median (IQR)	55.30 (23.98)	61.35 (25.16)	−6.05 (−15.22, 2.97)	*0.033^b^	62.10 (23.55)	59.88 (23.80)	60.11 (27.86)	49.22 (21.50)	0.309^f^

Variables	Hp 2-2				Non-Hp 2-2			
	Vitamin E	Placebo	Diff (95% CI)	*p* value	Vitamin E	Placebo	Diff (95% CI)	*p* value
(b) *Subgroup analysis of final measurements at 6 months with comparisons between haptoglobin genotype and Vitamin E and placebo group*								
Number (%)	42 (25.30)	44 (26.51)			42 (25.30)	38 (22.89)		
Time from baseline to completion visit days, Median (IQR)	176.50 (13.00)	177.00 (14.00)		0.595^b^	175.50 (12.00)	177.00 (14.00)		0.417^b^
*Physical measurements*								
BMI Kg/m^2^, mean (SD)	27.79 (5.33)	27.96 (5.36)	0.17 (−2.12, 2.46)	0.882^a^	27.30 (4.54)	26.39 (4.54)	−0.92 (−2.94, 1.11)	0.370^a^
Waist circumference cm, mean (SD)	91.78 (9.94)	95.17 (11.11)	3.39 (−1.14, 7.92)	0.140^a^	94.17 (10.57)	92.85 (11.30)	−1.31 (−6.18, 3.55)	0.592^a^
Systolic BP (Peripheral) mmHg, mean (SD)	133.21 (13.63)	128.92 (14.08)	−4.29 (−10.24, 1.65)	0.155^a^	133.33 (16.51)	130.42 (12.96)	−2.91 (−9.57, 3.74)	0.386^a^
Diastolic BP (Peripheral) mmHg, mean (SD)	74.61 (8.30)	72.78 (7.51)	−1.82 (−5.21, 1.57)	0.288^a^	73.75 (9.09)	70.99 (6.07)	−2.76 (−6.24, 0.72)	0.118^a^
*Metabolic parameters*								
HbA1c %, mean (SD)	7.79 (1.26)	8.29 (1.67)	0.49 (−0.14, 1.13)	0.127^a^	7.70 (1.13)	7.65 (0.80)	−0.05 (−0.49, 0.39)	0.820^a^
Total cholesterol mmol/L, Median (IQR)	4.30 (1.23)	4.20 (1.10)	−0.10 (−0.59, 0.39)^e^	0.473^b^	4.10 (0.93)	3.60 (1.25)	−0.50 (−0.94, −0.06)^e^	*0.016^b^
LDL cholesterol mmol/L, Median (IQR)	2.40 (1.13)	2.30 (0.80)	−0.10 (−0.52, 0.32)^e^	0.410^b^	2.30 (0.70)	1.90 (0.75)	−0.40 (−0.72, −0.08)^e^	*0.006^b^
HDL cholesterol mmol/L, Median (IQR)	1.10 (0.23)	1.10 (0.28)	0.00 (−0.10, 0.10)^e^	0.972^b^	1.10 (0.40)	1.10 (0.30)	0.00 (−0.15, 0.15) ^e^	0.478^b^
Triglycerides mmol/L, Median (IQR)	1.35 (0.75)	1.30 (1.25)	−0.05 (−0.49, 0.29)^e^	0.819^b^	1.40 (1.30)	1.10 (0.90)	−0.30 (−0.83, 0.23)^e^	0.110^b^
*Renal parameters*								
Creatinine μmol/L, mean (SD)	66.52 (18.89)	78.27 (24.62)	11.76 (0.74, 22.77)	*0.037^a^	77.03 (19.95)	78.94 (25.82)	1.90 (−9.95,13.75)	0.749^a^
eGFR ml/min/1.72m^2^, mean (SD)	97.49 (16.75)	85.18 (22.97)	−12.32 (−22.42,−2.22)	*0.017^a^	88.51 (16.99)	84.02 (20.06)	−4.48 (−14.02,5.06)	0.351^a^
ACR mg/mmol, Median (IQR)	1.25 (3.18)	1.75 (5.45)	0.50 (−1.53, 2.73)^e^	0.093^b^	2.15 (4.63)	2.90 (4.25)	0.75 (−0.46, 2.86)^e^	0.691^b^
*Haematological parameters*								
Haptoglobin mg/dL, mean (SD)	98.98 (44.44)	101.50 (39.12)	2.52 (−15.41, 20.46)	0.780^a^	120.36 (57.20)	132.26 (52.66)	11.91 (12.65,36.4)	0.337^a^
Ferritin μg/L, Median (IQR)	57.50 (70.00)	58.50 (107.00)	1.0 (−33.55, 37.55)^e^	0.701^b^	79.00 (94.00)	58.00 (90.00)	21.0 (−60.28,22.28)^e^	0.089^b^
Transferrin g/L, mean (SD)	2.51 (0.40)	2.46 (0.36)	−0.04 (−0.21, 0.12)	0.597^a^	2.35 (0.41)	2.46 (0.40)	0.11 (−0.07, 0.29)	0.228^a^
Fe Concentration μmol/L, mean (SD)	12.90 (4.87)	11.72 (4.68)	−1.18 (−3.59, 1.22)	0.329^a^	12.89 (5.73)	13.81 (4.07)	0.92 (−1.55, 3.40)	0.460^a^
Alpha-tocopherol, μg/ml,Median (IQR)	38.25 (38.04)	39.22 (33.56)	0.97 (−15.21,16.98)^e^	0.789^b^	52.04 (46.30)	45.53 (44.10)	−6.51 (−27.12,13.4)^e^	0.765^b^
*Vascular markers*								
hsCRP mg/L, Median (IQR)	1.90 (3.08)	1.4 (2.95)	−0.50 (−1.74, 0.74)^e^	0.441^b^	1.20 (1.93)	1.65 (2.43)	0.45 (−0.48, 1.48)^e^	0.390^b^
dROMS, Median (IQR)	293.50 (87.00)	270.5 (120.00)	−23.0 (−59.08, 25.08)^e^	0.267^b^	266.50 (85.00)	280.50 (115.0)	14.0 (−28.45,50.45)^e^	0.544^b^
BAPs uM, Median (IQR)	2218.0 (315.00)	2192.5 (393.00)	−25.5 (−169.9,105.87)^e^	0.717^b^	2252.5 (326.0)	2186.5 (418.0)	−66.0 (−206.8,87.8)^e^	0.965^b^
CIMT: Average of left and right mm, mean (SD)	0.66 (0.12)	0.67 (0.13)	0.01 (−0.05, 0.06)	0.754^a^	0.69 (0.15)	0.67 (0.12)	−0.02 (−0.08, 0.04)	0.579^a^
CIMT: Maximum of left and right mm, mean (SD)	0.80 (0.14)	0.81 (0.15)	0.01 (−0.06, 0.07)	0.839^a^	0.84 (0.18)	0.82 (0.15)	−0.02 (−0.09, 0.05)	0.556^a^
EndoPAT: LnRHI, mean (SD)	0.64 (0.26)	0.68 (0.23)	0.04 (−0.06, 0.15)	0.418^a^	0.69 (0.28)	0.61 (0.26)	−0.08 (−0.20, 0.04)	0.194^a^
EndoPAT: Augmentation index 75bpm%,mean (SD)	19.90 (16.66)	18.48 (13.23)	−1.43 (−7.86, 5.01)	0.660^a^	20.55 (18.53)	12.89 (9.80)	−7.65 (−14.36,−0.95)	*0.022^a^
Pulse Wave Analysis: Systolic BP mmHg, mean (SD)	119.62 (12.64)	115.95 (12.32)	−3.67 (−9.02, 1.69)	0.177^a^	118.33 (16.13)	114.58 (12.13)	−3.75 (−10.16,2.65)	0.247^a^
Pulse Wave Analysis: Diastolic BP mmHg,mean (SD)	77.83 (8.68)	76.59 (8.51)	−1.24 (−4.93, 2.45)	0.505^a^	77.79 (10.84)	74.66 (8.22)	−3.13 (−7.45, 1.19)	0.153^a^
Pulse Wave Velocity m/s, mean (SD)	8.34 (1.56)	8.51 (1.72)	0.17 (−0.54, 0.87)	0.641^a^	8.62 (1.61)	8.29 (1.55)	−0.32 (−1.03, 0.38)	0.362^a^
SphygmoCor: Augmentation index %, mean (SD)	30.85 (9.07)	29.48 (9.77)	−1.38 (−5.45, 2.70)	0.503^a^	28.60 (17.21)	29.00 (10.31)	0.41 (−6.00, 6.81)	0.900^a^
Oxidised LDL IU/L, Median (IQR)	62.10 (23.55)	59.88 (23.80)	−2.22 (−11.43,7.05)^e^	0.484^b^	60.11 (27.86)	49.22 (21.50)	−10.89 (−22.2,−0.75) ^e^	*0.012^b^

*DM* Diabetes mellitus, *BMI* body mass index, *BP* blood pressure, *eGFR* estimated glomerular filtration rate by CKD-EPI formula, *ACR* albumin creatinine ratio, *LDL-C* low density lipoprotein cholesterol, *HDL-C* high density lipoprotein cholesterol, *Fe* iron, *hsCRP* highly sensitive c-reactive protein, *dROMS* derivatives of reactive-oxygen species, *BAP* Biological antioxidant potential, *CIMT* carotid artery intima media thickness, *LnRHI* log reactive-hyperaemia index.

^a^Independent Sample T test.

^b^Mann–Whitney U test.

^c^Pearson Chi-Square test.

^d^Fisher Exact test.

^e^The median difference was derived by quantile regression.

^f^Interaction test between hp genotype and Vitamin E placebo group. *P < 0.05.

Subgroup analysis showed no improvement in the primary outcome of RHI-EndoPAT (*P* > 0.05; Table 2a) across the Hp genotypes. However, the non-Hp2-2 group had a higher RHI-EndoPAT-derived augmentation index (AIx@75bpm) in the Vit-E group when compared to placebo group, *p* = 0.022. When analysed as a change from baseline, the difference was still statistically significant (*p* < 0.05). No significant difference was seen in the Hp2-2 group, *p* > 0.05 (Table 2b).

Secondary Outcomes: The markers of inflammation, hsCRP and Hp concentrations, were similar in both Vit-E and placebo groups across the Hp genotypes (*p* > 0.05 for all). There was no significant difference in the oxidative stress markers: dROMS and BAPs between the Vit-E and placebo groups across the Hp genotypes, *p* > 0.05 (Table 2b). However, in the non-Hp2-2 group Vit-E supplementation led to a significantly higher total Cholesterol, LDL-C and ox-LDL-C (Vit E Group: median Cholesterol(IQR): 4.10(0.93); median LDL-C (IQR): 2.30 (1.13)mmol/L; median ox-LDL (IQR): 60.11(27.86)IU/L vs placebo: median cholesterol(IQR): 3.60(1.25); median LDL-C (IQR): 1.90 (0.75)mmol/L; median ox-LDL (IQR): 49.22(21.50)IU/L), *p* < 0.05. On further analysis in the non-Hp2-2 group of the change in the LDL-C from baseline we observed that there was a clinically insignificant change in LDL-C from baseline in the Vit-E Group with median change 0.0 (0.73) mmol/L vs placebo group −0.10 (0.60) mmol/L. However, in the Vit-E group, median Ox-LDL concentrations increased by 7.34(27.47) IU/L from baseline vs placebo 2.70(18.08) IU/L groups. Other vascular function physiological measurements of Sphygmocor- PWV and CIMT were similar in both vit-E and placebo groups across all Hp genotypes, *p* > 0.05.

Incidentally we found that the serum creatinine concentration was significantly lower and corresponding eGFR was higher in the Vit-E group compared to the placebo group in only the Hp2-2 individuals (Mean eGFR (SD): Vit-E group: 97.49 (16.75) mL/min per 1.73 m^2^: 85.18 (22.97) mL/min per 1.73 m^2^, *p* = 0.017. This effect was not seen in the non-Hp2-2 group (Mean eGFR (SD): Vit-E group: 88.51 (16.99) mL/min per 1.73 m^2^ vs Placebo: 84.02 (20.06) mL/min per 1.73 m^2^, *p* > 0.05 (Table 2). No differences in proteinuria was seen. Compared to baseline, serum ferritin concentrations at follow up significantly decreased in Vit-E group for both Hp2-2 (median difference (IQR): Vit E group: −12(28) μg/L; placebo: −1.50 (22) μg/L, *p* < 0.05) and non-Hp2-2 groups (median difference: Vit E group −18.00 (39) μg/; placebo: −2.00 (24) μg/L, *p* < 0.05). These outcomes of serum creatinine concentrations, eGFR and ferritin concentrations were not pre-specified as primary or secondary outcomes so these results would be considered exploratory.

We analysed the correlations of all outcomes with baseline alpha-tocopherol concentrations and final results adjusting for baseline alpha-tocopherol concentrations. Baseline alpha-tocopherol concentrations correlated positively only with baseline haptoglobin concentrations (*p* < 0.05). The results of the final outcomes were similar to unadjusted analysis (Supplementary Table 1a, b).

### Non pre-specified post-hoc analysis

We hypothesised that lower baseline haptoglobin concentrations would be associated with improvement in measurements of inflammation, oxidative stress and vascular function. Lower baseline haptoglobin concentrations (regardless of genotype) was associated with an improvement in hsCRP (β = −0.03, *p* = 0.002) and dROMS (β = −0.34, *p* = 0.002) even after adjustment for other variables in a multivariable model (inclusive of age, gender, BMI and haptoglobin genotype). Further analysis showed that at a sensitivity and specificity of >50%, the optimal cut off for baseline haptoglobin concentration at which >20% decline in hsCRP and >10% decline in dROMS was seen was at ≤119 mg/dl. Approximately 62.79% (54/86) in the Hp2-2 group had Hp ≤ 119 mg/dl when compared to 40% (32/80) in the non-Hp2-2 group. (Supplementary Table 2a, b). We further did an interaction test in the main analysis using haptoglobin concentrations ≤119 mg/dl and >119 mg/dl. We found a positive interaction for RHI-EndoPAT-derived augmentation index (AI@75bpm) in that a detrimental effect was seen in individuals with haptoglobin >119 mg/dl whereas no statistically significant effect was seen in the individuals with haptoglobin ≤ 119 mg/dl (Supplementary Table 2c).

## Discussion

In our primary analysis, we did not observe a significant beneficial effect of Vit-E supplementation on inflammation, oxidative stress or vascular function in both the haptoglobin genotypes. Previous studies have shown that it has preferential benefits in the Hp2-2 genotype individuals in terms of cardiovascular complications in diabetes^6,7^. This is in contrast with the results of a smaller crossover study of 20 subjects wherein an improvement in forearm vascular resistance and blood flow was seen within 8 weeks in the Hp2-2 individuals^12^. Our results are similar to a previous double blind RCT of 89 T2DM subjects where a high dose of Vit-E (1800 IU) was for a duration of 12 months and no significant change in vascular dilator function was seen^19^. Our study had a larger sample size than the above studies and the duration of Vit-E supplementation was 6 months.

Possible reasons for these contradictory results from previous outcome based trials: (1) We have analysed the systemic pathway of vascular dysfunction and endothelial dysfunction, whereas the gene dependent effect of Vit-E supplementation maybe more at the cellular level, for example myocardial fibrosis or cardiac vasculature in particular. Future studies need to assess cardiac function and physiology in detail. (2) The population studied included the multi-ethnic groups of Chinese, Indians and Malays, whereas most previous studies have been conducted in Europeans. In our population Indians were more likely to have the Hp2-2 genotype and this group of individuals are also at the highest risk for cardiovascular complications. It is possible that there are inherent differences in lipid and metabolic profiles in these individuals which may have contributed to the negative results. The effect of ethnicity needs to be studied in greater details. (3) It is believed that unlike statins and antihypertensives, the primary mechanism of action of antioxidants may be prevention of new lesions. Hence, ideally they would need to be used for more than 5 years to show a demonstrable benefit^20^. It is likely that we need further optimisation of Vit-E treatment in terms of dose and duration to optimise the alpha-tocopherol concentrations before we see significant effects of vascular function. In a recent long-term prospective cohort study, higher serum α-tocopherol biochemical status has been associated with lower risk of overall mortality^21^.

We observed a harmful effect in the non-Hp2-2 group in terms of increased arterial stiffness measured as RHI-EndoPAT-derived AIx. This measurement is known to correlate with brachial artery pulse wave velocity and may be a function of arterial elasticity and stiffness^15^. Most epidemiological and animal studies in the past have shown no effect of Vit- E on vascular function in the non-Hp2-2 individuals. One study has reported an adverse effect of Vit-E on HDL function in the non-Hp2-2 type 1 diabetes patients^22^. In fact in our study we saw a parallel increase in ox-LDL even relative to LDL-C change in this subgroup. There was a strong interaction effect seen in the overall group with Hp > 119 mg/dl. This deleterious effect of Vit-E exerts raises the question of possible pro-oxidant effect of Vit-E in the presence of sufficient quantitative (concentrations) and qualitative (non-Hp2-2) haptoglobin in these individuals^23^. This phenomenon of pro-oxidant effect of Vit-E under different oxidant conditions has been described previously^24^ and needs to be studied in greater details in presence of different concentrations of haptoglobin.

We used the dose of 400 IU of supplemental Vit-E daily. Meta-analyses have shown that high doses (≥400 IU/day) has been associated with an increase in mortality and is not recommended for primary or secondary prevention^25,26^. The effect of lower doses of supplementation or using other forms of supplementation (gamma-, delta-tocopherol and tocotrienols) which has shown to be beneficial^27^ needs to be studied.

We observed that Vit-E impacted eGFR in the Hp2-2 group only. Hp2-2 genotype individuals have been observed to have a higher risk of renal function decline when compared to the non-Hp2-2 group which may be attributed to higher ferritin deposition in the kidneys^28^. Vit- E supplementation did lead to a significant decrease in ferritin concentrations (change from baseline) in both the Hp2-2 and non-Hp2-2 groups when compared to placebo. This change has likely impacted the Hp2-2 group more due to a relatively inefficient haemoglobin scavenging system and more antioxidant effect when compared to a possible pro-oxidant effect in Non-Hp2-2. This observation has to be considered exploratory as this was not a pre-specified primary or secondary outcome.

We have previously seen that endothelial cell apoptosis in patients with diabetes was associated with haptoglobin concentrations rather than the genotypes^29^. Hence, we performed a subgroup analysis to see whether the response to treatment is dependent on the haptoglobin concentrations. We found that lower baseline haptoglobin concentrations (regardless of genotype) (≤119 mg/dl) was associated with an improvement in a marker of inflammation, hsCRP and an oxidative stress marker dROMS even after adjustment for other variables in a multivariable model. It has also been seen that individuals with Hp2-2 genotype tend to have lower haptoglobin concentrations than non-Hp2-2 genotype individuals^30^. In our study as well the haptoglobin levels were significantly lower in the Hp2-2 individuals. However, it is important to note that up to 40% of patients in the non-Hp2-2 group also had low haptoglobin concentrations at baseline and a similar number (approx. 37%) had high concentrations in the Hp2-2 group which may have resulted in dilution of the effects seen. It is possible that further personalised supplementation based on the haptoglobin concentrations may be beneficial.

Some limitations of our study has been the compliance rate, the variance in alpha-tocopherol concentrations in the individuals and the duration of the study (<5 years). Although the compliance rate was satisfactory in more than 70% of the individuals there was a variability in the alpha-tocopherol concentrations and change from baseline amongst the individuals suggesting variable absorption and bioavailability. Another limitation was that 73% of the non-Hp2-2 group had one Hp2 allele (ie. Hp1-2 rather than Hp1-1 genotype) hence reducing the difference from the Hp2-2 group. We used the RHI-EndoPAT which measures only the dynamic reactivity and although may better reflect short term changes due to interventions^31^ does not correlate as well with brachial artery flow mediated dilatation as this measurement does not only reflect nitric oxide but also may related with endothelial derived prostaglandins and endothelium-derived hyperpolarising factor in hypertensive patients^15^. In balance we did supplement this with the Sphygmocor based measurements which measures baseline pulse-wave-velocity and EndoPAT based augmentation index a reflection of arterial stiffness and elasticity.

The main strengths of this study is the relatively larger sample size and relatively longer duration compared to studies currently reported. We evaluated vascular function comprehensively in terms of oxidative stress, inflammation and physiological vascular function. Our study was completed in a real world setting and the results may therefore be generalised to our population.

## Conclusion

In conclusion, we did not see a preferential benefit or deleterious effect of Vit-E supplementation on inflammation, oxidative stress or vascular function in the multi-ethnic Singapore patients with Hp2-2 genotype. As an exploratory analysis, incidentally we observed that Vit-E supplementation led to an improvement in renal function in the Hp2-2 genotype. We found preliminary evidence suggesting a possible deleterious effect of an increase in arterial stiffness with Vit-E supplementation in individuals with haptoglobin concentrations >119 mg/dl. The possible mechanism of the deleterious effect on these individuals needs to be studied.

Future studies should consider personalisation based on baseline haptoglobin concentrations in patients with T2DM rather than just Hp2-2 genotype to evaluate impact on the detailed lipid pathways, cardiac and renal physiology. The impact of ethnic differences needs to be explored in greater detail.

## Supplementary information

Supplementary Appendix

## Data Availability

The datasets are available from the corresponding author on reasonable request.

## References

[1] Sarwar N (2010). Diabetes mellitus, fasting blood glucose concentration, and risk of vascular disease: a collaborative meta-analysis of 102 prospective studies. Emerging Risk Factors Collaboration. Lancet.

[2] Rask-Madsen C, King GL (2007). Mechanisms of Disease: endothelial dysfunction in insulin resistance and diabetes. Nat. Clin. Pr. Endocrinol. Metab..

[3] Levy AP (2010). Haptoglobin: basic and clinical aspects. Antioxid. Redox Signal.

[4] Asleh R (2003). Genetically determined heterogeneity in haemoglobin scavenging and susceptibility to diabetic cardiovascular disease. Circ. Res..

[5] Vardi M, Blum S, Levy AP (2012). Haptoglobin genotype and cardiovascular outcomes in diabetes mellitus-natural history of the disease and the effect of vitamin E treatment. Meta-analysis of the medical literature. Eur. J. Intern. Med.

[6] Blum S (2010). Vitamin E reduces cardiovascular disease in individuals with diabetes mellitus and the haptoglobin 2–2 genotype. Pharmacogenomics.

[7] Dalan R, Goh LL (2018). The protean role of haptoglobin and haptoglobin genotypes on vascular complications in diabetes mellitus. Eur. J. Prev. Cardiol..

[8] Alshiek JA (2017). Anti-oxidative treatment with vitamin E improves peripheral vascular function in patients with diabetes mellitus and haptoglobin 2-2 genotype. Diab. Res. Clin. Pr..

[9] Saha N, Ong YW (1984). Distribution of haptoglobins in different dialect groups of Chinese, Malays and Indians in Singapore. Ann. Acad. Med. Singap..

[10] Levey AS (2009). CKD-EPI (Chronic Kidney Disease Epidemiology Collaboration). A new equation to estimate glomerular filtration rate. Ann. Intern. Med..

[11] Soejima M, Koda Y (2008). TaqMan-based real-time PCR for genotyping common polymorphisms of haptoglobin (HP1 and HP2). Clin. Chem..

[12] Rubinshtein R (2010). Assessment of endothelial function by non-invasive peripheral arterial tonometry predicts late cardiovascular adverse events. Eur. Heart J..

[13] Bonpei T, Yuko H (2013). Disparity between EndoPat measurement and brachial artery flow-mediated vasodilatation in hypertensive patients. JACC.

[14] Vassalle C (2008). An easy and reliable automated method to estimate oxidative stress in the clinical setting. Methods Mol. Biol..

[15] Holvoet P, Stassen JM, Van Cleemput J, Collen D, Vanhaecke J (1998). Oxidized low density lipoproteins in patients with transplant‐associated coronary artery disease. Arterioscler Thromb. Vasc. Biol..

[16] Criockshank K (2002). Aortic pulse wave velocity and its relationship to mortality in diabetes and glucose intolerance: an integrated index of vascular function?. Circulation.

[17] Touboul PJ, Hennerici MG, Meairs S (2007). Mannheim Carotid intima-media thickness consensus (2004–2006). An update on behalf of the Advisory Board of the 3rd and 4th Watching the Risk Symposium, 13th and 15th European Stroke Conferences, Mannheim, Germany, 2004, and Brussels, Belgium, 2006. Cerebrovasc. Dis..

[18] Aversa A (2008). Chronic administration of sildenafil improves markers of endothelial function in men with type 2 diabetes. Diabet. Med..

[19] Economides PA (2005). The effect of vitamin E on endothelial function of micro- and macrocirculation and left ventricular function in type 1 and type 2 diabetic patients. Diabetes.

[20] Steinberg D (1995). Clinical trials of antioxidants in atherosclerosis: are we doing the right thing?. Lancet.

[21] Huang J (2019). Relationship between serum alpha-tocopherol and overall and cause-specific mortality. Circ. Res..

[22] Costacou T, Levy AP, Miller RG (2016). Effect of vitamin E supplementation on HDL function by haptoglobin genotype in type 1 diabetes: results from the HapE randomized crossover pilot trial. Acta Diabetol..

[23] Bowry VW, Stocker R (1993). Tocopherol-mediated peroxidation. The prooxidant effect of vitamin E on the radical-initiated oxidation of human low-density lipoprotein. J. Am. Chem. Soc..

[24] Miyazawa T, Burdeos GC, Itaya M, Nakagawa K (2019). Vitamin E: regulatory redox interactions. IUBMB Life.

[25] Miller ER (2005). Meta-analysis: high dosage vitamin E supplementation may increase all-cause mortality. Ann. Intern. Med.

[26] Bjelakovic G, Nikolova D, Gluud LL, Simonetti RG, Gluud L (2012). Antioxidant supplements for prevention of mortality in healthy participants and patients with various diseases. Cochrane Database Syst. Rev..

[27] Jiang Q (2014). Natural forms of vitamin E: metabolism, antioxidant, and anti-inflammatory activities and their role in disease prevention and therapy. Free Radic. Biol. Med..

[28] Costacou T (2018). Is magnetic resonance imaging detection of kidney iron deposition increased in haptoglobin 2-2 genotype carriers with type 1 diabetes?. Antioxid. Redox Signal.

[29] Dalan R, Liu X, Goh LL, Bing S, Luo KQ (2017). Endothelial cell apoptosis correlates with low haptoglobin concentrations in diabetes. Diab. Vasc. Dis. Res..

[30] Langlois MR, Delanghe JR (1996). Biological and clinical significance of haptoglobin polymorphism in humans. Clin. Chem..

[31] Schnabel RB (2011). Noninvasive vascular measurement in the community: cross-sectional relations and comparison of methods. Circ. Cardiovasc Imaging.

