# Supplementary material for: Impact of Vitamin E supplementation on vascular function in haptoglobin genotype stratified diabetes patients (EVAS Trial): a randomised controlled trial
Source: Nutr Diabetes. 2020 Apr 27;10:13. doi: 10.1038/s41387-020-0116-7 (PMC7186220; doi:10.1038/s41387-020-0116-7)
Supplement: Supplementary file 1 — Supplementary Appendix [file 41387_2020_116_MOESM1_ESM.docx]

Supplementary Appendix

Supplementary Table 1A: Final Measurements at 6 months with comparisons between haptoglobin genotype and Vitamin E and placebo group adjusted for baseline alpha-tocopherol level

| **Variables** | **Overall** | | | **Hp 2-2** | | | **Non Hp 2-2** | | |
| --- | --- | --- | --- | --- | --- | --- | --- | --- | --- |
|  | **Vitamin E** | **Placebo** | ***p* value** | **Vitamin E** | **Placebo** | ***p* value** | **Vitamin E** | **Placebo** | ***p* value** |
| **Physical measurements** |  |  |  |  |  |  |  |  |  |
| Final BMI Kg/m^2^ | 27.54  (26.46, 28.61) | 27.24  (26.16, 28.33) | 0.704^a^ | 27.71  (26.07, 29.36) | 28.04  (26.44, 29.64) | 0.780^a^ | 27.30  (25.90, 28.71) | 26.39  (24.91, 27.87) | 0.375 ^a^ |
| Final Waist circumference cm | 92.97  (90.65, 95.29) | 94.10  (91.75, 96.45) | 0.501^a^ | 91.84  (88.58, 95.11) | 95.11  (91.92, 98.30) | 0.159 ^a^ | 94.18  (90.81, 97.55) | 92.84  (89.30, 96.38) | 0.588 ^a^ |
| Final Systolic BP (Peripheral) mmHg | 133.25  (130.16, 136.34) | 129.64  (126.51, 132.77) | 0.107^a^ | 133.23  (128.94, 137.52) | 128.91  (124.72, 133.10) | 0.157 ^a^ | 133.36  (128.76, 137.95) | 130.40  (125.57, 135.23) | 0.379 ^a^ |
| Final Diastolic BP (Peripheral) mmHg | 4.30  (4.28, 4.32) | 4.27  (4.25, 4.30) | 0.088^b^ | 74.57  (71.13, 77.02) | 72.82  (70.43, 75.21) | 0.312 ^a^ | 4.29  (4.26, 4.33) | 4.26  (4.23, 4.29) | 0.140^b^ |
| **Metabolic parameters** |  |  |  |  |  |  |  |  |  |
| Final HbA1c % | 7.75  (7.47, 8.02) | 7.99  (7.71, 8.27) | 0.230^a^ | 7.81  (7.36, 8.27) | 8.27  (7.82, 8.71) | 0.163 ^a^ | 7.65  (7.33, 7.97) | 7.70  (7..39, 8.00) | 0.824 ^a^ |
| Final Total cholesterol mmol/L | 4.33  (4.13, 4.52) | 4.06  (3.87, 4.26) | 0.061^a^ | 4.41  (4.14, 4.67) | 4.26  (4.00, 4.52) | 0.450 ^a^ | 4.25  (3.97, 4.53) | 3.83  (3.53, 4.12) | *0.041 ^a^ |
| Final LDL cholesterol mmol/L | 2.47  (2.31, 2.63) | 2.23  (2.06, 2.39) | 0.035^a^ | 2.57  (2.35, 2.79) | 2.38  (2.15, 2.61) | 0.230 ^a^ | 2.37  (2.14, 2.59) | 2.06  (1.82, 2.29) | 0.060 ^a^ |
| Final HDL cholesterol mmol/L | 1.13  (1.06, 1.19) | 1.13  (1.06, 1.19) | 0.980^a^ | 1.12  (1.04, 1.19) | 1.11  (1.04, 1.18) | 0.963 ^a^ | 1.14  (1.03, 1.03) | 1.14  (1.03, 1.26) | 0.949 ^a^ |
| Final Triglycerides mmol/L | 1.71  (1.43, 2.00) | 1.69  (1.40, 1.97) | 0.898^a^ | 0.43  (0.25, 0.60) | 0.33  (0.15, 0.51) | 0.474^b^ | 1.84  (1.44, 2.23) | 1.40  (0.99, 1.82) | 0.138 ^a^ |
| **Renal parameters** |  |  |  |  |  |  |  |  |  |
| Final Creatinine μmol/L | 71.59  (65.85, 77.33) | 78.69  (73.09, 84.30) | 0.082^a^ | 66.27  (58.29, 74.26) | 78.50  (70.76, 86.24) | *0.032 ^a^ | 77.20  (68.84, 85.56) | 78.78  (70.55, 87.00) | 0.789 ^a^ |
| Final ACR mg/mmol | 4.96  (0.87, 9.04) | 7.98  (3.80, 12.16) | 0.308^a^ | 3.27  (-2.20, 8.73) | 8.79  (3.32, 14.25) | 0.161 ^a^ | 6.57  (0.19, 12.95) | 7.30  (0.60, 14.00) | 0.875 ^a^ |
| **Haematological parameters** |  |  |  |  |  |  |  |  |  |
| Final Haptoglobin mg/dL | 109.88  (99.13, 120.63) | 115.54  (104.66, 126.42) | 0.466^a^ | 98.97  (86.03, 111.92) | 101.51  (88.86, 114.15) | 0.782 ^a^ | 120.23  (103.39, 137.07) | 132.40  (114.70, 150.11) | 0.324 ^a^ |
| Final Ferritin μg/L | 91.24  (74.49, 108.00) | 85.43  (68.47, 102.39) | 0.631^a^ | 74.40  (51.07, 97.74) | 84.62  (61.82, 107.41) | 0.536 ^a^ | 107.92  (83.61, 132.22) | 86.57  (61.01, 112.12) | 0.232 ^a^ |
| Final Transferrin g/L | 2.43  (2.34, 2.51) | 2.46  (2.38, 2.55) | 0.566^a^ | 2.50  (2.39, 2.62) | 2.47  (2.36, 2.58) | 0.688 ^a^ | 2.35  (2.23, 2.48) | 2.46  (2.33, 2.59) | 0.231 ^a^ |
| Final Fe Concentration μmol/L | 12.89  (11.69, 14.09) | 12.75  (11.53, 13.98) | 0.877^a^ | 12.91  (11.19, 14.64) | 11.71  (10.02, 13.40) | 0.322 ^a^ | 12.86  (111.18, 14.53) | 13.84  (12.06, 15.62) | 0.423 ^a^ |
| Final Haemoglobin g/dL | 12.41  (11.54, 13.28) | 12.88  (12.05, 13.72) | 0.432^a^ | 12.86  (11.64, 14.07) | 12.78  (11.47, 14.10) | 0.930 ^a^ | 12.84  (11.40, 14.28) | 12.11  (10.67, 13.56) | 0.456 ^a^ |
| Final Alpha-tocopherol concentration, μg/ml | 50.04  (44.91, 55.16) | 46.67  (41.48, 51.86) | 0.363^a^ | 45.42  (39.02, 51.81) | 41.33  (35.08, 47.48) | 0.367 ^a^ | 55.02  (46.85, 63.19) | 52.45  (43.86, 61.03) | 0.667 ^a^ |
| **Vascular markers** |  |  |  |  |  |  |  |  |  |
| Final hsCRP mg/L | 2.82  (1.82, 3.82) | 2.77  (1.76, 3.78) | 0.947^a^ | 2.56  (1.00, 4.12) | 3.14  (1.61, 4.67) | 0.600 ^a^ | 0.20  (-0.17, 0.58) | 0.37  (-0.02, 0.76) | 0.544^b^ |
| Final Oxidative Stress index: dROMS | 281.40  (266.30, 296.50) | 276.96  (261.67, 292.24) | 0.684^a^ | 292.88  (271.50, 314.27) | 275.61  (254.72, 296.50) | 0.255 ^a^ | 270.19  (248.31, 292.06) | 278.21  (255.22, 301.21) | 0.616 ^a^ |
| Final Oxidative Stress index: BAP uM | 2226.71  (2167.66, 2285.77) | 2215.26  (2155.49, 2275.03) | 0.788^a^ | 2219.09  (2137.99, 2300.18) | 2177.96  (2098.75, 2257.18) | 0.474 ^a^ | 2237.32  (2150.42, 2324.22) | 2255.15  (2163.79, 2346.51) | 0.779 ^a^ |
| Final CIMT: Average of left and right mm | 0.67  (0.65, 0.70) | 0.67  (0.64, 0.70) | 0.883^a^ | 0.66  (0.62, 0.70) | 0.67  (0.63, 0.71) | 0.690 ^a^ | 0.69  (0.65, 0.73) | 0.67  (0.63, 0.72) | 0.535 ^a^ |
| Final CIMT: Maximum of left and right mm | 0.82  (0.79, 0.85) | 0.81  (0.78, 0.85) | 0.788^a^ | 0.80  (0.76, 0.85) | 0.81  (0.77, 0.85) | 0.770 ^a^ | 0.84  (0.79, 0.89) | 0.82  (0.77, 0.87) | 0.523 ^a^ |
| Final EndoPAT : Endothelial function - LnRHI | 0.66  (0.61, 0.72) | 0.65  (0.59, 0.70) | 0.701^a^ | 0.64  (0.56, 0.71) | 0.68  (0.61, 0.76) | 0.401 ^a^ | 0.69  (0.61, 0.77) | 0.61  (0.52, 0.70) | 0.195 ^a^ |
| Final EndoPAT : Augmentation index at 75bpm % | 2.81  (2.62, 3.01) | 2.55  (2.36, 2.75) | 0.061^b^ | 20.17  (15.58, 24.76) | 18.23  (14.75, 22.71) | 0.550 ^a^ | 2.76  (2.47, 3.06) | 2.34  (2.03, 2.64) | *0.050^b^ |
| Final Pulse Wave Analysis: Systolic BP (Central) mmHg | 118.98  (116.10, 121.87) | 115.31  (112.39, 118.23) | 0.079^a^ | 119.74  (115.89, 123.59) | 115.84  (112.08, 119.60) | 0.154 ^a^ | 118.34  (113.89, 122.78) | 114.58  (109.90, 119.25) | 0.249 ^a^ |
| Final Pulse Wave Analysis: Diastolic BP (Central) mmHg | 77.80  (75.84, 79.77) | 75.70  (73.71, 77.69) | 0.140^a^ | 77.85  (75.19, 80.52) | 76.57  (73.97, 79.17) | 0.496 ^a^ | 77.79  (74.80, 80.79) | 74.65  (71.50, 77.80) | 0.154 ^a^ |
| Final Pulse Wave Analysis: Pulse Wave Velocity m/s | 8.48  (8.13, 8.82) | 8.41  (8.06, 8.76) | 0.803^a^ | 8.37  (7.86, 8.87) | 8.49  (7.99, 8.98) | 0.735 ^a^ | 8.62  (8.15, 9.09) | 8.29  (7.79, 8.78) | 0.327 ^a^ |
| Final SphygmoCor: Augmentation index % | 29.75  (27.14, 32.35) | 29.22  (26.60, 31.84) | 0.780^a^ | 30.76  (27.81, 33.70) | 29.57  (26.73, 32.41) | 0.567 ^a^ | 28.55  (24.21, 32.89) | 29.05  (24.49, 33.62) | 0.874 ^a^ |
| Final Oxidised LDL, IU/L | 63.65  (59.65, 67.64) | 58.06  (54.02, 62.11) | 0.054^a^ | 63.45  (58.11, 68.80) | 61.07  (55.85, 66.29) | 0.528 ^a^ | 63.73  (57.64, 69.82) | 54.71  (48.31, 61.11) | *0.045 ^a^ |

^a^ANCOVA adjusted for covariate alpha- tocopherol level; ^b^Transformation (Ln X) were done as the assumption homogeneity of variance was found not fulfilled; *p value<0.05

DM: Diabetes mellitus, BMI:body mass index, BP: blood pressure, eGFR: estimated glomerular filtration rate by CKD-EPI formula, ACR: albumin creatinine ratio, LDL-C: Low density lipoprotein cholesterol, HDL-C: high density lipoprotein cholesterol Fe: iron, hsCRP: highly sensitive c-reactive protein, dROMS: derivatives of reactive oxygen species, BAPs: Biological anti-oxidant potential, CIMT: carotid artery intima media thickness, LnRHI: log reactive hyperemia index.

Supplementary Table 1B: Relationship between baseline variables with alpha tocopherol concentrations

|  | **Adj. Coeff** | **95% CI** | ***p* value** |
| --- | --- | --- | --- |
| **Physical measurements** |  |  |  |
| BMI Kg/m^2^ | -0.015 | -0.040, 0.011 | 0.252 |
| Waist circumference cm | -0.003 | -0.057, 0.050 | 0.907 |
| Systolic BP (Peripheral) mmHg | -0.008 | -0.086, 0.069 | 0.830 |
| Diastolic BP (Peripheral) mmHg | -0.017 | -0.058, 0.023 | 0.401 |
| HbA1c % | 0.006 | -0.000, 0.012 | 0.063 |
| **Lipid parameters** |  |  |  |
| Total cholesterol mmol/L | -0.003 | -0.008, 0.001 | 0.169 |
| LDL cholesterol mmol/L | -0.003 | -0.007, 0.001 | 0.200 |
| HDL cholesterol mmol/L | 0.0003 | -0.001, 0.002 | 0.723 |
| Triglycerides mmol/L | -0.004 | -0.011, 0.003 | 0.277 |
| Creatinine μmol/L | -0.039 | -0.132, 0.054 | 0.409 |
| eGFR ml/min/1.73 m^2^ | 0.049 | -0.049, 0.147 | 0.329 |
| Urine ACR mg/mmol | -0.106 | -0.243, 0.030 | 0.126 |
| **Haematological parameters** |  |  |  |
| Haptoglobin mg/dL | 0.309 | 0.049, 0.569 | 0.020* |
| Ferritin μg/L | -0.073 | -0.484, 0.337 | 0.724 |
| Transferrin g/L | -0.000 | -0.002, 0.002 | 0.801 |
| Fe Concentration μmol/L | -0.001 | -0.030, 0.027 | 0.921 |
| Haemoglobin g/dL | 0.002 | -0.008, 0.012 | 0.713 |
| **Vascular markers** |  |  |  |
| hsCRP mg/L | 0.003 | -0.015, 0.021 | 0.740 |
| Oxidative Stress index: dROMS | -0.318 | -0.650, 0.014 | 0.060 |
| Oxidative Stress index: BAPs uM | -0.746 | -2.221, 0.730 | 0.320 |
| CIMT: Average of left and right mm | -0.001 | -0.001, 0.000 | 0.086 |
| CIMT: Maximum of left and right mm | -0.001 | -0.001, 0.000 | 0.174 |
| EndoPAT : Endothelial function - LnRHI | 0.000 | -0.001, 0.002 | 0.704 |
| EndoPAT : Augmentation index at 75bpm % | 0.001 | -0.071, 0.074 | 0.970 |
| Sphygmocor Systolic BP (Central) mmHg | -0.011 | -0.083, 0.060 | 0.758 |
| Sphygmocor Diastolic BP (Central) mmHg | -0.007 | -0.055, 0.040 | 0.757 |
| Sphygmocor Pulse Wave Velocity m/s | -0.007 | -0.016, 0.001 | 0.099 |
| SphygmoCor: Augmentation index % | 0.033 | -0.017, 0.083 | 0.193 |
| Oxidised LDL IU/L | 0.005 | -0.110, 0.119 | 0.938 |

Multiple linear regression adjusted for age, gender and ethnicity,p *<0.05

Supplementary Table 2A: Multivariable model analysing the change in hsCRP concentrations with baseline haptoglobin concentrations adjusted for age, gender, BMI, haptoglobin genotype and alpha-tocopherol concentrations at follow up.

| **Change in hsCRP** | **Coefficient** | **p-value** | **[95% Conf. Interval]** | |
| --- | --- | --- | --- | --- |
| Haptoglobin concentration | -0.027 | *0.002 | -0.045 | -0.01 |
| Alpha-tocopherol concentration at follow up | 0.005 | 0.75 | -0.025 | 0.035 |
| Age | 0.02 | 0.632 | -0.063 | 0.103 |
| Gender |  |  |  |  |
| Male | reference |  |  |  |
| Female | 0.382 | 0.652 | -1.287 | 2.051 |
| Ethnicity |  |  |  |  |
| Chinese | reference |  |  |  |
| Malay | -0.205 | 0.865 | -2.575 | 2.165 |
| Indian | -0.111 | 0.917 | -2.214 | 1.992 |
| BMI | 0.045 | 0.609 | -0.129 | 0.22 |
| Non-hp22 | reference |  |  |  |
| Hp22 | -1.064 | 0.241 | -2.847 | 0.72 |

HsCRP: High sensitive C-reactive protein; Hp2-2: Haptoglobin 2-2 genotype individuals; Non Hp2-2: non Haptoglobin 2-2 genotype individuals. *p value <0.05

Supplementary Table 2B: Multivariable model analysing the change in dROMS (derivatives of reactive oxygen metabolites) concentrations with baseline haptoglobin concentrations adjusted for age, gender, BMI, haptoglobin genotype and alpha-tocopherol concentrations at follow up.

| **Change in dROMs** | **Coefficient** | **p-value** | **[95% Conf.** **Interval]** | |
| --- | --- | --- | --- | --- |
| Haptoglobin concentration | -0.34 | 0.002 | -0.55 | -0.13 |
| Alpha-tocopherol concentration at follow up | 0.301 | 0.11 | -0.068 | 0.67 |
| Age | 0.198 | 0.698 | -0.811 | 1.208 |
| Gender |  |  |  |  |
| Male | reference |  |  |  |
| Female | 14.371 | 0.166 | -6.045 | 34.788 |
| Ethnicity |  |  |  |  |
| Chinese | reference |  |  |  |
| Malay | -20.095 | 0.172 | -48.989 | 8.8 |
| Indian | -8.509 | 0.513 | -34.153 | 17.136 |
| BMI | 2.769 | 0.011 | 0.636 | 4.901 |
| Non-*hp22* | reference |  |  |  |
| *Hp22* | -10.34 | 0.349 | -32.098 | 11.417 |

*Hp2-2*: Haptoglobin 2-2 genotype individuals,

Non *Hp2-2*: nonHaptoglobin 2-2 genotype individuals, *p value <0.05

Supplementary Table 2C: The interaction effect of Vitamin E intervention with Haptogloglobin concentrations on all outcome variables

|  | **Hp ≤ 119** | | **Hp > 119** | | ***p* value**^d^ |
| --- | --- | --- | --- | --- | --- |
|  | **Vitamin E (n=46)** | **Placebo (n=42)** | **Vitamin E (n=37)** | **Placebo (n=40)** |  |
| Number (%) |  |  |  |  |  |
| Time from baseline to completion visit days, Median (IQR) | 176.00 (9.00) | 179.00 (12.00) | 175.00 (15.00) | 176.50 (14.00) |  |
| **Physical measurements** |  |  |  |  |  |
| BMI Kg/m^2^, mean (SD) | 26.05 (4.56) | 26.94 (4.66) | 29.36 (4.80) | 27.54 (5.43) | 0.076 |
| Waist circumference cm, mean (SD) | 92.21 (10.91) | 93.99 (11.63) | 93.90 (9.50) | 94.21 (10.84) | 0.662 |
| Systolic BP (Peripheral) mmHg, mean (SD) | 134.41 (16.02) | 132.44 (14.90) | 131.90 (13.86) | 126.65 (11.31) | 0.461 |
| Diastolic BP (Peripheral) mmHg, mean (SD) | 75.73 (9.02) | 74.05 (7.30) | 72.30 (7.92) | 69.75 (5.75) | 0.714 |
| **Metabolic parameters** |  |  |  |  |  |
| HbA1c %, mean (SD) | 7.65 (1.06) | 8.20 (1.51) | 7.86 (1.34) | 7.77 (1.18) | 0.103 |
| Total cholesterol mmol/L, Median (IQR) | 4.15 (1.35) | 4.15 (1.35) | 4.10 (1.10) | 3.90 (1.35) | 0.259 |
| LDL cholesterol mmol/L, Median (IQR) | 2.40 (0.88) | 2.10 (1.13) | 2.25 (0.75) | 2.10 (0.93) | 0.158 |
| HDL cholesterol mmol/L, Median (IQR) | 1.10 (0.33) | 1.05 (0.25) | 1.10 (0.40) | 1.10 (0.30) | 0.405 |
| Triglycerides mmol/L, Median (IQR) | 1.25 (1.33) | 1.30 (1.30) | 1.50 (0.80) | 1.20 (0.80) | 0.402 |
| **Renal parameters** |  |  |  |  |  |
| Creatinine μmol/L, mean (SD) | 75.06 (18.71) | 76.80 (20.52) | 68.20 (20.93) | 80.18 (28.61) | 0.211 |
| eGFR ml/min/1.72m^2^, mean (SD) | 93.42 (18.42) | 84.56 (18.16) | 92.72 (16.43) | 84.67 (24.25) | 0.909 |
| ACR mg/mmol, Median (IQR) | 1.30 (3.41) | 2.90 (6.30) | 2.05 (3.60) | 1.75 (3.58) | 0.089 |
| **Haematological parameters** |  |  |  |  |  |
| Haptoglobin mg/dL, mean (SD) | 74.80 (30.28) | 87.14 (35.81) | 151.87 (40.23) | 145.80 (40.71) | 0.109 |
| Ferritin μg/L, Median (IQR) |  |  |  |  | 0.278 |
| Transferrin g/L, mean (SD) | 2.38 (0.40) | 2.51 (0.36) | 2.49 (0.42) | 2.42 (0.38) | 0.097 |
| Fe Concentration μmol/L, mean (SD) | 13.50 (4.64) | 13.27 (4.11) | 12.25 (5.94) | 12.27 (4.80) | 0.883 |
| Alpha-tocopherol, μg/ml,Median (IQR) | 39.57 (43.13) | 37.11 (40.06) | 48.95 (40.05) | 45.64 (37.02) | 0.846 |
| **Vascular markers** |  |  |  |  |  |
| hsCRP mg/L, Median (IQR) | 0.90 (1.65) | 0.90 (2.40) | 2.35 (4.10) | 2.10 (2.85) | 0.05 |
| dROMS, Median (IQR) | 259.00 (87.00) | 262.50 (82.00) | 293.50 (74.00) | 286.00 (125.00) | 0.302 |
| BAPs uM, Median (IQR) | 2234.50 (351.00) | 2148.50 (347.00) | 2209.50 (370.00) | 2225.50 (283.00) | 0.866 |
| CIMT: Average of left and right mm, mean (SD) | 0.68 (0.14) | 0.66 (0.10) | 0.67 (0.14) | 0.68 (0.15) | 0.525 |
| CIMT: Maximum of left and right mm, mean (SD) | 0.83 (0.16) | 0.80 (0.12) | 0.82 (0.16) | 0.82 (0.17) | 0.536 |
| EndoPAT: LnRHI, mean (SD) | 0.66 (0.26) | 0.63 (0.23) | 0.66 (0.29) | 0.67 (0.27) | 0.677 |
| EndoPAT: Augmentation index 75bpm%,mean (SD) | 18.28 (16.65) | 18.81 (13.57) | 22.58 (18.46) | 12.83 (9.37) | 0.028* |
| Pulse Wave Analysis: Systolic BP mmHg, mean (SD) | 119.78 (16.00) | 118.10 (12.87) | 118.00 (12.37) | 112.40 (10.80) | 0.345 |
| Pulse Wave Analysis: Diastolic BP mmHg,mean (SD) | 79.41 (10.21) | 78.36 (7.95) | 75.87 (8.94) | 72.90 (7.99) | 0.489 |
| Pulse Wave Velocity m/s, mean (SD) | 8.48 (1.65) | 8.71 (1.62) | 8.48 (1.52) | 8.09 (1.62) | 0.212 |
| SphygmoCor: Augmentation index %, mean (SD) | 30.22 (8.81) | 27.71 (10.55) | 29.11 (18.09) | 30.88 (9.16) | 0.257 |
| Oxidised LDL IU/L, Median (IQR) | 65.79 (26.69) | 56.83 (24.15) | 56.44 (24.23) | 54.25 (24.40) | 0.245 |

^a^Independent Sample T test; ^b^Mann Whitney U test; ^c^The median difference was derived by quantile regression. ^d^Interaction test between hp genotype and Vitamin E placebo group.

DM: Diabetes mellitus, BMI:body mass index, BP: blood pressure, eGFR: estimated glomerular filtration rate by CKD-EPI formula, ACR: albumin creatinine ratio, LDL-C: Low density lipoprotein cholesterol, HDL-C: high density lipoprotein cholesterol Fe: iron, hsCRP: highly sensitive c-reactive protein, dROMS: derivatives of reactive oxygen species, BAPs: Biological anti-oxidant potential, CIMT: carotid artery intima media thickness, LnRHI: log reactive hyperemia index.
